# Evaluation of patients presenting with febrile seizures in an Iranian referral hospital: emphasis on the frequency of meningitis and co-infections

**DOI:** 10.1186/s12887-023-04120-z

**Published:** 2023-06-22

**Authors:** Mahsa Soti Khiabani, Mahya sadat Mohammadi, Mahmoud Reza Ashrafi, Syeda Bushra Haider, Syeda Iqra Haider, Shima Mahmoudi, Setareh Mamishi

**Affiliations:** 1 Children’s Medical Center, Pediatrics Center of Excellence, Tehran, Iran; 2 Pediatric Emergency Department, Tehran University of Medical Sciences, Tehran, Iran; 3 Pediatric Neurology Department, Tehran University of Medical Sciences, Tehran, Iran; 4 Tehran University of Medical Sciences, Tehran, Iran; 5 Pediatric Infectious Disease Research Center, Tehran University of Medical Science, , Children’s Medical Center Hospital, Dr. Gharib Street, Keshavarz Boulevard, I.R Tehran, Iran; 6 Biotechnology Centre, Silesian University of Technology, Gliwice, 44-100 Poland; 7 Department of Infectious Diseases, Pediatrics Center of Excellence, Children’s Medical Center, Tehran University of Medical Sciences, Tehran, Iran

**Keywords:** Febrile seizure, CSF, Meningitis, Covid-19, Lumbar puncture

## Abstract

**Introduction:**

Febrile seizures are one of the most common diseases that physicians encounter in pediatric emergency departments. Two important aspects of managing patients presenting with a febrile seizure are meningitis exclusion and co-infection investigation. This study was designed to determine any infection that occurs concomitantly with a febrile seizure episode and also to assess the frequency of meningitis among children presenting with febrile seizures.

**Methods:**

This retrospective cross-sectional study was conducted at the Children’s Medical Center, an Iranian pediatric referral hospital. All patients aged 6 months to 5 years presenting with febrile seizures from 2020 to 2021 were included. Patients’ data were collected from the medical report files. The presence of respiratory, gastrointestinal, and urinary infections was evaluated. Moreover, the detection of SARS-CoV-2 using reverse transcription polymerase chain reaction (RT-PCR) was performed for suspicious cases.

The results of urine and stool analysis, as well as blood, urine, and stool cultures were checked. The frequency of lumbar puncture (LP) performance and its results were studied. The relationship between white blood cells (WBC), erythrocyte sedimentation rate (ESR), and C-reactive protein in meningitis was evaluated.

**Results:**

A total of 290 patients were referred to the Children’s Medical Center, Tehran, Iran, due to fever and seizures. The mean age of the patients was 21.5 ± 13.0 months, and 134 (46.2%) were female. Out of 290 patients, 17% presented with respiratory infections. Nasopharyngeal SARS**-**CoV**-**2 RT**-**PCR was requested for 50 patients (17%), of which nine (3%) were reported positive and two patients had multi-inflammatory syndrome in children (MIS-C). Fever without local signs, gastroenteritis, and urinary tract infections were found in 40%, 19%, and 14% of the patients, respectively. LP was requested for 97 participants (33.4%) to evaluate central nervous system infection, of which 22 cases were suggestive of aseptic meningitis. Among laboratory tests, leukocytosis was significantly related to aseptic meningitis (odds ratio = 11.1, 95% CI = 3.0- 41.5). The blood culture testing result was positive in seven patients; all of them were due to skin contamination.

**Conclusion:**

Evaluation of patients for possible meningitis is necessary for febrile seizure management. Although the prevalence of bacterial meningitis in these patients is not high, according to this study and other studies conducted in Iran, aseptic meningitis, especially after Measles, Mumps, and Rubella (MMR) vaccination should be considered. Leukocytosis and increased CRP can predict the occurrence of aseptic meningitis in these patients. However, further studies with a larger sample size are highly recommended. Moreover, during the COVID-19 pandemic, it is recommended to pay attention to an acute COVID-19 infection or evidence of MIS-C in children with fever and seizure.

## Introduction

Febrile seizures are defined as seizures that occur with a temperature of 38° Celsius or higher that are not due to a central nervous system (CNS) infection or any metabolic disturbance, and the absence of any history of a prior afebrile seizure. The incidence of febrile seizures is estimated at 2–5% in European and American children, and it is commonly seen in children between 6 months and 5 years old [1].

Febrile seizures pose a significant challenge for pediatricians because of the recurrence and high incidence. One important aspect of managing these patients is meningitis exclusion because fever and convulsions can also occur in the clinical course of meningitis. Studies show that 1 in 4 children with bacterial meningitis will present with a seizure [2].

According to the American Academy of Pediatrics guidelines, lumbar puncture (LP) should be performed to rule out meningitis if the child is ill-appearing or there are clinical signs or symptoms of concern. For children between 6-12 months of age for whom *Haemophilus influenzae* type b and *Streptococcus pneumoniae* immunization has not been performed or is uncertain, LP may be an option. For children who have been treated with antibiotics, cerebrospinal fluid (CSF) analysis should be strongly considered because antibiotic therapy might mask meningitis signs and symptoms [3].

The other aspect of febrile seizure patient management is searching for co-infections. Seizures may happen during illnesses such as a cold, respiratory infections, gastroenteritis, or an ear infection. The most common cause of fever is a viral infection, which is reported in approximately 80% of the patients. Viral infections that are commonly associated with febrile seizures include influenza, adenovirus, parainfluenza, and herpesvirus-6 (roseola infantum) [4–6]. The most common reported bacterial infection is otitis media [4]; therefore, correct and timely diagnosis and treatment of these concurrent infections is necessary because it controls the symptoms and prevents further complications.

Since the onset of the COVID-19 pandemic worldwide, some studies reported the association between febrile seizures and COVID-19 and suggested considering COVID-19 infection in children hospitalized with febrile seizures [7, 8].

This study was designed to determine any infection that occurs concomitantly with a febrile seizure, including respiratory tract infections, gastroenteritis, urinary tract infections, and occult bacteremia. In addition, we assessed the association between COVID-19 infection and febrile seizures. The other aim of this study was to evaluate the frequency of LP performance, CSF analysis in children presenting with fever and seizures.

## Methods

In this retrospective cross-sectional study, 290 patients referred to the Children’s Medical Center Hospital in Iran between 2020 and 2021 were included.

Children’s Medical Center, an Iranian referral pediatrics hospital, has a pediatric emergency department with 60 beds. At first, patients presenting with febrile illness and seizures are admitted to the emergency department. After evaluation and pediatric specialist assessment, if the patient does not have red flags signs and symptoms, they are discharged; if red flags exist, more diagnostic and therapeutic measures are taken, and then they are transferred to the appropriate subspecialty ward.

Red flag signs and symptoms in a child presenting with febrile seizures include complex febrile seizures (defined as the seizure that has one or more of the following features: lasts longer than 15 min, focal seizure or > one seizure within 24 h); meningeal signs such as a positive Kernig’s sign and/or a positive Brudzinski sign and/or neck stiffness; altered level of consciousness for more than one hour after the interruption of the febrile seizures; evolving non-blanching rashes in an ill child; bulging anterior fontanelle; tachycardia out of proportion to body temperature or tachycardia that persists even after normalization body temperature; signs of moderate to severe respiratory distress, such as tachypnea, grunting, and low oxygen saturation (< 92% on air), and chest wall recessions [6].

The information recorded in the patient’s hospital file was studied. Inclusion criteria were patients with a fever > 38 °C and seizures between the ages of 6 months and 5 years. Exclusion criteria were seizure mimickers, electrolyte disturbance, and a prior history of a febrile seizures.

The study was ethically approved by Tehran University of Medical Sciences (IR.TUMS.CHMC.REC.1400.096 and IR.TUMS.CHMC.REC.1400.097) and conducted in accordance with the Code of Ethics of the World Medical Association (Declaration of Helsinki) for experiments on humans.

Laboratory test results, including complete blood count (CBC) and white blood cell differential, erythrocyte sedimentation rate (ESR), C-reactive protein (CRP), blood culture, urine analysis, and urine culture, were collected. The urine sample was taken in the midstream at first, and if it was abnormal in the initial analysis (WBC > 5 per high powered field (hpf)), positive for nitrite, positive for bacteria, or positive for leukocyte esterase), a sterile sample was taken to confirm urinary tract infection. We assessed patients who were subjected to LP to rule out bacterial or viral meningitis and the results of CSF analysis, and culture were studied. Blood culture results were studied to identify any occult bacteremia.

The presence of respiratory, gastrointestinal, and urinary symptoms in patients was evaluated. To find co-infections, the results of the urine analysis, urine cultures, chest X-ray, detection of SARS-CoV-2 using reverse transcription polymerase chain reaction (RT-PCR), and stool exam and culture were analyzed.

### Statistical analysis

All statistical analyses were performed using SPSS (Statistical Package for the Social Sciences) version 13.0 software (SPSS Inc). Categorical variables were reported as frequency and percentages. Normally distributed continuous variables were presented as means with standard deviations. Fisher exact tests or χ2 tests were used to compare categorical variables between different groups. A *p* value < 0.05 was predetermined as the level of significance. Odds ratios (OR) were calculated with 95% confidence intervals.

## Results

A total of 290 patients were referred to the Children’s Medical Center, Tehran, Iran due to fever and seizures during 2020 and 2021. The mean age of the patients was 21.5 ± 13.1 months, and 134 (46.2%) were female. Among all cases, 231 (79.7%) presented with simple seizures, while 59 (20.3%) had complex febrile seizures.

**Fig. 1:**
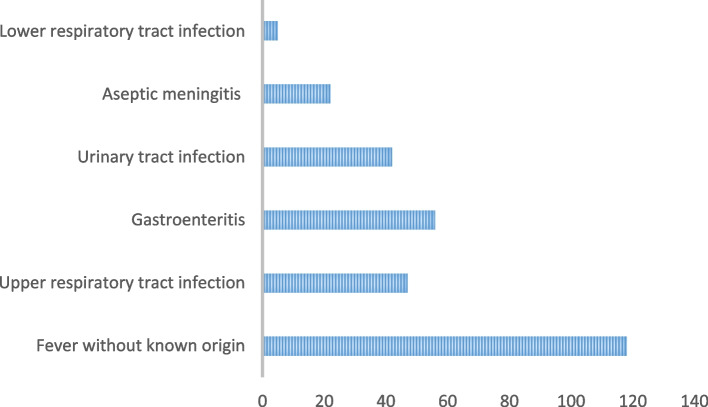
Infectious sources in patients with fever and seizure

Out of 290 patients, 52 presented with respiratory symptoms (cough, coryza, and breathing problems). Among them, 47 patients had an upper respiratory infection, while in five patients, the lower respiratory system was involved (Fig. 1).

Considering that this study was conducted during the COVID-19 pandemic, nasopharyngeal SARS-CoV-2 RT-PCR was requested for 50 (17%) patients, of which 9 (3%) cases were reported as positive. Further diagnostic investigations in the case of two patients illustrated that their fever and seizure were caused by multi-inflammatory syndrome in children (MIS-C).

Gastrointestinal symptoms, including vomiting, diarrhea, and abdominal pain were found in 115 patients (39%) with febrile seizures. Gastroenteritis was the final diagnosis of 56 (19%) patients; among them, 31 cases had an abnormal stool exam (bacterial colitis, WBC or RBC > 5 per high powered field). In total, 73 cases presented with other gastrointestinal symptoms like nausea, vomiting, and loss of appetite.

Positive stool cultures were reported in 4 patients (*Shigella dysenteriae*, Salmonella (Group D), *Candidia albicans*, and *Shigella flexneri*. Out of the 290 patients, 155 (59.6%) had normal urine analysis (WBC 0–5/hpf and RBC 0–4/hpf), while 42 (16.2%) had active urine analysis results (WBC more than 5/hpf and RBC more than 4/hpf). Positive urine culture was reported in 20 cases (12 cases with mixed bacterial growth, 2 cases with *Escherichia coli*, 2 cases with *Klebsiella* spp., 2 cases with skin contamination, 1 case with *Enterococcus faecium*, and 1 case *E. faecalis*).

In 118 patients, despite diagnostic investigations, no specific infectious source for fever was found. Lumbar puncture was performed in 97 patients (33.4%) to evaluate CNS infection, of which 22 cases were suggestive of aseptic meningitis. In other patients, CSF analysis was normal.

In 31 patients, fever and seizures occurred after the Measles, Mumps, and Rubella (MMR) vaccination, and in 18 cases, post-vaccination aseptic meningitis was confirmed through cerebrospinal fluid analysis. In addition to these 18 cases, 4 other patients were also diagnosed with aseptic meningitis based on the CSF analysis criteria (positive CSF viral profile with Enterovirus in two cases). The results showed no significant relationship between aseptic meningitis and elevated ESR (*p* value = 0.54). However, leukocytosis was significantly related to aseptic meningitis (odds ratio = 11.1, 95% CI = 3.0- 41.5).

A blood culture was done for all patients who presented with a febrile seizure. In seven cases the culture was reported positive, all of which was due to skin contamination.

## Discussion

In a patient presenting with fever and convulsions, the assessment of meningitis is one of the most important aspects of patient management. Indications for CSF assessment are always a challenging topic for pediatricians. The frequency of performing LP in this study was 33%, and the prevalence of meningitis was 7%, all of which were aseptic meningitis.

The prevalence of bacterial meningitis among children with fever and convulsions has been reported to be very diverse. The prevalence of LP performance in this study is higher compared to similar studies conducted in other countries (7% in Saudi Arabia, and 13–16% in Japan) [9, 10]. In some studies, only complex febrile seizure patients have been examined, and the prevalence of performing LP was 25% [11]. The absence of immunization for *Streptococcus pneumoniae* in Iran immunization protocol causes higher LP performance for children younger than 12 months [12].

In a study conducted in Pakistan, 157 patients with the first episode of febrile seizures were studied, and the frequency of meningitis was 12% [13]. In another study performed in France, 205 patients (6–11 months) with the first simple febrile seizure were evaluated. LP was performed for 21%, and no case of meningitis was found [14]. In a study done in Saudi, 1375 febrile seizure cases were evaluated. LP was performed for 7%, and the prevalence of meningitis was 0.9% [9]. In a similar study performed in Iran in 800 patients with febrile seizure, LP was performed for 56% of them. The prevalence of meningitis and bacterial meningitis was 10% and 0.6%, respectively [15]. It is important to keep in mind that these studies were conducted in different communities in terms of vaccination, ages, and statistical populations in terms of simple and complex seizures. The summary of these studies is given in Table 1.

**Table 1:** LP performance and its results in febrile seizure patients in different studies

Author	Year	Country	Total number (n)	Patient group	LP performance n (%)	Meningitis n (%)	Aseptic meningitis n (%)	Bacterial meningitis n (%)
Siddiqui HB. [13]	2012-2013	Pakistan	157	aged 6 to 60 months with first episode of febrile seizures	Not mentioned	12 (7.6)	Not mentioned	12 (7.6)
Guedj R. [14]	2007-2011	France	205	aged 6 to 11 months with simple febrile seizure	61 (29)	Not mentioned	Not mentioned	0
Eldardear A [9]	2015-2019	Saudi Arabia	1375	all ages with febrile seizures	108 (7)	9 (0.6)	Not mentioned	Not mentioned
Heydarian F. [15]	2005-2010	Iran	800	aged 6 to 18 months with febrile seizure	453 (56)	80 (10)	Not mentioned	5 (60)
Lee J. [11]	2007-2014	United States	28810	aged 0 to 5 years with complex febrile seizure	7445 (20)	80 (30)	Not mentioned	Not mentioned
Current study	2020-2021	Iran	219	aged 6 to 60 months with febrile seizure	97 (33)	22 (7)	22 (7)	0

Previous studies have shown that aseptic meningitis can occur after MMR vaccination, so fever and seizures can be part of the clinical presentation of aseptic meningitis in these patients [16–18]. A recent study in Iran shows an increase in aseptic meningitis after the first MMR vaccination [19]. Therefore, aseptic meningitis is one of the main differential diagnoses in a child who presents with fever and seizures up to 6 weeks after MMR vaccination. An increase in inflammatory markers and leukocytosis can be a sign of aseptic meningitis in these patients.

Another aspect of managing febrile seizure patients is co-infection investigation. According to previous studies among viruses, roseola infantum (exanthem subitum), influenza A, human coronavirus HKU1, herpesvirus 6, adenovirus, parainfluenza, and varicella are the most prevalent causes. Other current co-infections with febrile seizures are middle ear infections, upper and lower airway infections (such as tonsillitis, sinusitis, bronchitis, and pneumonia), tooth infections, and gastroenteritis (especially those caused by rotavirus) [20–23]. According to our study, the most common infections are gastroenteritis (19%), respiratory tract infection (17%), and UTI (14%). In 40% of cases, no infectious source was found for the fever. Considering that not treating some of these infections can be associated with serious complications, it is important to obtain a detailed history and complete physical examination.

At the beginning of the COVID-19 pandemic, several cases of acute COVID-19 infection and MIS-C were reported from Iran’s pediatric community. The COVID-19 infection can cause fever, respiratory, or digestive symptoms in children, and following this fever, febrile convulsion can occur [24–29]. According to this, febrile seizures can be seen in COVID-19 infections.

MIS-C is a rare condition associated with SARS-CoV-2 that usually occurs 2–6 weeks after a child is infected with SARS-CoV-2. MIS-C causes inflammation in different organs, including the heart, lungs, kidneys, brain, skin, eyes, or gastrointestinal tract. Fever is considered one of the main criteria of this disease, and it occurs along with an increase in inflammatory markers, evidence of COVID-19 infection, and involvement of at least two mentioned organs. Seizures can be a sign of central nervous system involvement in these patients, so febrile seizures can be part of the manifestations of the MIS-C in children [30]. In our study, nine had positive nasopharynx SARS-CoV-2 RT-PCR. In 2 patients, after clinical evaluation, a MIS-C diagnosis was proposed. Previous studies showed 0.5% of COVID-19 subjects were diagnosed with febrile seizures [7, 8]; therefore, searching for traces of COVID-19 infection in patients with fever and seizures seems logical.

To our knowledge, we evaluated the patients presenting with febrile seizures in an Iranian referral hospital during the COVID-19 pandemic. However, this study has some limitations. Due to the fact that this study was done retrospectively, the history and physical examination of some patients may not have been accurately mentioned in the hospital files, so conducting prospective studies about co-infections in febrile seizure patients can be very helpful.

## Conclusion

Evaluation of patients in terms of meningitis is necessary for febrile seizure management. Although the prevalence of bacterial meningitis in these patients is not high, according to this study and other studies conducted in Iran, aseptic meningitis, especially after MMR vaccination should be considered. Leukocytosis and increased CRP can predict the occurrence of aseptic meningitis in these patients. However, further studies with a larger sample size are highly recommended. Moreover, during the COVID-19 pandemic, it is recommended to pay attention to an acute COVID-19 infection or evidence of MIS-C in children with fever and seizure.

## Data Availability

The data that support the findings of this study are available from the corresponding author upon reasonable request.

## Abbreviations

COVID-19: Coronavirus disease 19
SARS-CoV-2: Severe acute respiratory syndrome coronavirus 2
RT-PCR: Reverse transcription polymerase chain reaction
MIS-C: Multisystem inflammatory syndrome in children
Hib: *Haemophilus influenzae* type b
CSF: Cerebrospinal fluid
LP: Lumbar Puncture
MMR: Measles, Mumps, and Rubella
